# Timing the Emergence of Resistance to Anti-HIV Drugs with Large Genetic Barriers

**DOI:** 10.1371/journal.pcbi.1000305

**Published:** 2009-03-13

**Authors:** Pankhuri Arora, Narendra M. Dixit

**Affiliations:** 1 Department of Chemical Engineering, Indian Institute of Science, Bangalore, India; 2 Bioinformatics Center, Indian Institute of Science, Bangalore, India; Imperial College London, United Kingdom

## Abstract

New antiretroviral drugs that offer large genetic barriers to resistance, such as the recently approved inhibitors of HIV-1 protease, tipranavir and darunavir, present promising weapons to avert the failure of current therapies for HIV infection. Optimal treatment strategies with the new drugs, however, are yet to be established. A key limitation is the poor understanding of the process by which HIV surmounts large genetic barriers to resistance. Extant models of HIV dynamics are predicated on the predominance of deterministic forces underlying the emergence of resistant genomes. In contrast, stochastic forces may dominate, especially when the genetic barrier is large, and delay the emergence of resistant genomes. We develop a mathematical model of HIV dynamics under the influence of an antiretroviral drug to predict the waiting time for the emergence of genomes that carry the requisite mutations to overcome the genetic barrier of the drug. We apply our model to describe the development of resistance to tipranavir in *in vitro* serial passage experiments. Model predictions of the times of emergence of different mutant genomes with increasing resistance to tipranavir are in quantitative agreement with experiments, indicating that our model captures the dynamics of the development of resistance to antiretroviral drugs accurately. Further, model predictions provide insights into the influence of underlying evolutionary processes such as recombination on the development of resistance, and suggest guidelines for drug design: drugs that offer large genetic barriers to resistance with resistance sites tightly localized on the viral genome and exhibiting positive epistatic interactions maximally inhibit the emergence of resistant genomes.

## Author Summary

The ability of HIV to rapidly acquire mutations responsible for resistance to administered drugs underlies the failure of current antiretroviral therapies for HIV infection. The recent advent of drugs that offer large genetic barriers to resistance, e.g., tipranavir and darunavir, presents a new opportunity to devise therapies that remain efficacious over extended durations. The large number of mutations that HIV must accumulate for resistance to drugs with large genetic barriers impedes the failure of therapy. Further, these drugs appear to exhibit activity against viral strains resistant to other drugs in the same drug class, thereby significantly improving options for therapy. Rational identification of treatment protocols that maximize the impact of these new drugs requires a quantitative understanding of the process whereby HIV overcomes large genetic barriers to resistance. We develop a model that describes HIV dynamics under the influence of a drug that offers a large genetic barrier to resistance and predict the time of emergence of viral strains that overcome the large barrier. Model predictions provide insights into the roles of various evolutionary forces underlying the development of resistance, quantitatively describe the development of resistance to tipranavir *in vitro*, and suggest guidelines for drug design.

## Introduction

Current antiretroviral therapies for HIV infection often fail to elicit lasting virological responses in patients because of the emergence of multidrug resistant strains of HIV [1],[2]. The enormous replication rate and the high mutation and recombination rates of HIV [3]–[7] propel the acquisition of mutations that confer upon HIV resistance to administered drugs. The same mutations are often responsible for resistance to multiple drugs belonging to a given drug class [1],[2]. Consequently, treatment options for patients who experience failure of therapy are restricted [8],[9]. The newly approved protease inhibitors (PIs), tipranavir and darunavir, offer large genetic barriers to resistance [10],[11]. The genetic barrier of a drug, *n*, is the number of mutations that HIV must accumulate to gain high level resistance to the drug [12]. When *n* is small (e.g., *n* = 1 for 3TC [1]), drug resistant genomes are likely to exist in patients prior to the onset of therapy [13]. As *n* increases, the likelihood of the pre-existence of resistant genomes decreases considerably [13],[14]. Resistant genomes must then emerge during therapy through mutation and/or recombination of susceptible genomes. The replication of susceptible genomes, however, is suppressed during therapy. Besides, HIV must undergo a large number of replication cycles to accumulate all the mutations required for resistance to a drug with large *n*. Consequently, the development of resistance to a drug with large *n* may be significantly delayed. Indeed, up to 9 months were required for HIV to develop resistance to tipranavir in *in vitro* serial passage experiments [10].

Current treatment guidelines for HIV infection recommend a combination of 3, but at least 2, active drugs, (i.e., drugs for which resistance has not developed) in order partly to increase the overall genetic barrier of therapy [9]. For treatment naïve patients, a combination of 2 nucleoside/nucleotide reverse transcriptase inhibitors (NRTIs) is typically employed in combination with either a non-nucleoside reverse transcriptase inhibitor (NNRTI), usually efavirenz, or a ritonavir-boosted PI, usually lopinavir [9]. With ritonavir-boosted lopinavir monotherapy, fewer patients achieved plasma HIV RNA levels below detection and more patients witnessed emergence of PI resistance mutations than in patients receiving ritonavir-boosted lopinavir in combination with 2 NRTIs [15]. Similarly, despite comparable times to virological failure, patients receiving a 2 drug combination of efavirenz and lopinavir experienced more frequent emergence of resistance than patients receiving a 3 drug combination of efavirenz or lopinavir and 2 NRTIs [16]. Therapy with 4 NRTIs had a similar response to therapy with efavirenz and 2 NRTIs [17]. Consequently, a 3 drug combination is the current standard of care for treatment naïve patients. When failure did occur with a 3 drug combination, it was typically associated with NNRTI resistance in patients receiving efavirenz but not with PI resistance in patients receiving lopinavir [16], in accordance with the larger genetic barriers offered by PIs than by NNRTIs [18]. The large genetic barrier in conjunction with a superior pharmacokinetic profile may also underlie the high rates of viral suppression despite sub-optimal adherence in patients receiving ritonavir-boosted lopinavir-based therapy [19].

For second-line therapy, which follows the failure of the initial regimen, a drug from a new drug class is recommended in order to minimize the risk of cross-resistance [9]. Thus, among several newly available agents [20], the fusion inhibitor enfuvirtide and the recently approved integrase inhibitor raltegravir present potent options. Both enfuvirtide and raltegravir, however, offer small genetic barriers and are therefore recommended for use in conjunction with a supporting drug such as darunavir [9]. Remarkably, the new PIs, tipranavir and darunavir, elicit responses against viral strains resistant to other PIs [11],[21], increasing options for second-line therapy. The new PIs thus present promising weapons to avert the failure of antiretroviral therapy. Indeed, significant efforts are ongoing to identify treatment protocols that maximize the impact of the new PIs [8],[22]. Identification of improved protocols hinges on our understanding of HIV dynamics under the influence of drugs that offer large genetic barriers to resistance and of the process by which HIV surmounts these large genetic barriers.

Description of the development of resistance to a drug with a large *n* is complicated for several reasons. First, resistance to such a drug typically develops gradually, increasing progressively with the number of mutations accumulated [10],[23]. As a result, the emergence and the competitive dynamics of a large number of distinct viral genomes carrying different combinations of resistance mutations and possessing various intermediate levels of resistance must be described. For instance, the accumulation of mutations at 6 loci confers high level resistance to tipranavir [10]. Consequently, depending on whether each resistance locus carries a mutation or not, 2^6^, or 64, distinct strains (see below) may emerge in the course of infection. Because HIV is diploid, the 64 strains yield 64 homozygous and 2016 different kinds of heterozygous virions, whose evolutionary dynamics must be followed to describe how the genetic barrier of tipranavir is overcome. Second, the population size of HIV *in vivo* may be small, especially under the influence of therapy, which implies that the emergence of resistant genomes is likely to be governed by stochastic rather than deterministic effects [24]. Third, in addition to mutation, recombination can play a significant role in the formation of drug resistant strains that carry multiple mutations [25],[26]. The influence of recombination, which is yet to be fully understood, depends on several factors, viz., the frequency of multiple infections of cells, the effective population size of HIV *in vivo*, and the nature of fitness interactions between resistance mutations, characterized by epistasis [27]–[33]. No models exist that describe HIV dynamics under the simultaneous influence of mutation, multiple infections of cells, recombination, epistatic interactions between multiple resistance mutations, and stochastic effects of finite population sizes. Consequently, timing the failure of antiretroviral drugs with large genetic barriers is currently not possible. Rational identification of improved treatment protocols is therefore precluded.

Here, we develop a model of HIV dynamics that quantitatively predicts the expected waiting time for the emergence of genomes that carry the requisite mutations for resistance to a drug with any given genetic barrier. Extant models of HIV dynamics assume that deterministic forces are predominant in the emergence of drug resistance [34]–[36]. Consequently, extant models predict that drug resistant genomes emerge immediately upon the initiation of therapy, albeit in small numbers. In contrast, especially when the genetic barrier is large, stochastic forces are expected to dictate the emergence of resistant genomes. A key consequence of the predominance of stochastic forces is a delay in the emergence of resistant genomes following the initiation of therapy. Our model accounts for this delay in a deterministic manner by predicting the expected waiting time for the emergence of resistant genomes. Model predictions capture the development of resistance to tipranavir *in vitro* quantitatively, indicating that our model captures the underlying dynamics of the development of resistance to antiretroviral drugs. Further, model predictions provide insights into the impact of underlying evolutionary forces on the development of drug resistance and suggest guidelines for drug design.

## Results

### Model Formulation

**Figure 1:**
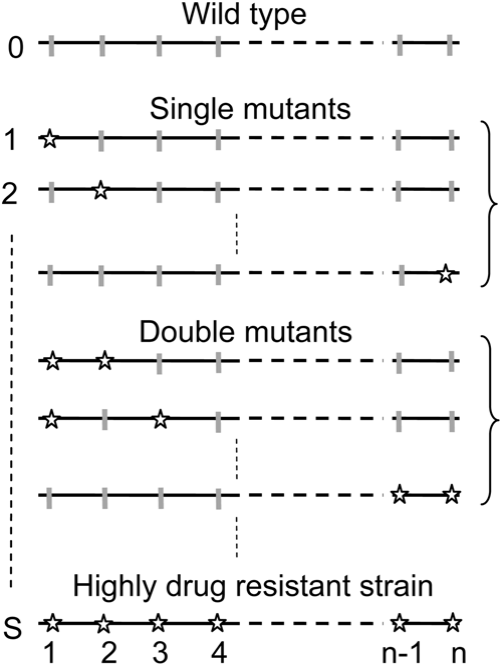
Schematic representation of the *S* viral genomes carrying different combinations of resistance mutations (stars) that emerge during the development of resistance to a drug with a genetic barrier *n*.

**Figure 2:**
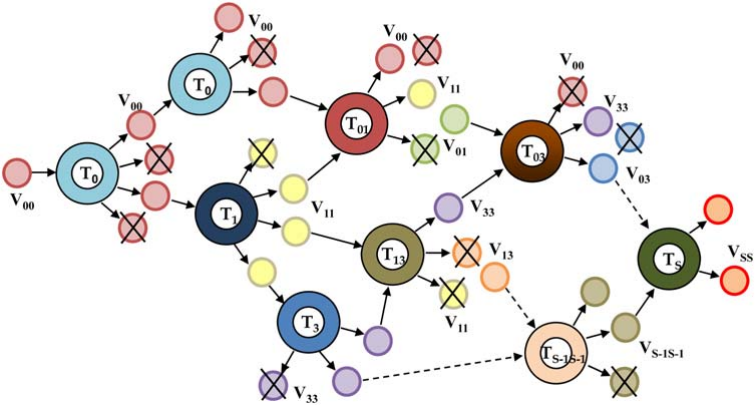
Schematic representation of the infection network indicating the various singly and doubly infected cells, *T_i_* and *T_ij_*, and homozygous and heterozygous virions, *V_ii_* and *V_ij_*, respectively, that emerge during the development of drug resistance. Non-infectious virions are crossed.

We consider uninfected cells, *T*, exposed in the presence of a PI with a genetic barrier *n* to a viral population, *V*, containing genomes highly susceptible to the PI. The highly susceptible, or wild-type, genomes are assumed to contain no resistance mutations. As infection proceeds, error-prone replication gives rise to mutant genomes. 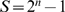 distinct mutant genomes can arise, each with at least one resistance mutation (Figure 1). Our aim is to determine the waiting time for the first formation of the genome that carries all the *n* resistance mutations and is therefore highly resistant to the drug. We number the different viral genomes 0, 1, 2, 3…*S*, where genome 0 represents the wild-type (Figure 1). We let *V_jh_* denote the population of virions containing genomes *j* and *h*, where *j*, *h* ∈ {0, 1, 2…*S*}. Because virions *V*_10_, for instance, are indistinguishable from virions *V*_01_, we impose the constraint *j*≤*h* [37]. Following the infection of a cell by a virion *V_jh_*, mutation and recombination give rise to a proviral genome *i* ∈ {0, 1, 2…*S*} with probability *Q_i_*(*jh*). We distinguish infected cells by the proviral genomes they contain: Cells *T_i_* are infected by a single provirus *i* and cells *T_ij_* by proviruses *i* and *j*, where *i*≤*j* and *i*, *j* ∈ {0, 1, 2…*S*}. Infected cells produce progeny virions. Drug action causes some of the progeny virions to be non-infectious [3],[34],[35]; we denote the noninfectious virion population by 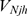. Cells *T_i_* and *T_ii_* infected by a single kind of provirus produce homozygous virions *V_ii_* and 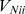. Cells *T_ij_* infected with distinct proviruses (*i*≠*j*) yield homozygous virions *V_ii_*, 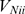, *V_jj_* and 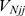 and heterozygous virions *V_ij_* and 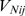. The resulting infection network is shown in part in Figure 2.

We construct dynamical equations to predict the time-evolution of various cell and viral populations and estimate the average waiting times for the first production of each of the *S* mutant proviral genomes (Methods). We denote by *W* the waiting time for the emergence of the provirus that contains all the *n* resistance mutations and hence overcomes the genetic barrier of the drug.

### Model Predictions

We solve model equations to describe the development of resistance in *in vitro* serial passage experiments (e.g., [10]). Here, *T*_0_ uninfected cells are exposed to viruses in the presence of a known concentration of the PI. Infection is allowed to progress until time *t_p_* (∼3.5 days), the duration of a passage. The resulting viral population is employed to initiate infection of a fresh set of *T*_0_ uninfected cells in the next passage. At the start of the first passage, the viral population is assumed to consist of *V*_00_ wild type viruses, highly susceptible to the drug. Gradually, genomes with increasing levels of drug resistance emerge.

#### Cell and virus dynamics

**Figure 3:**
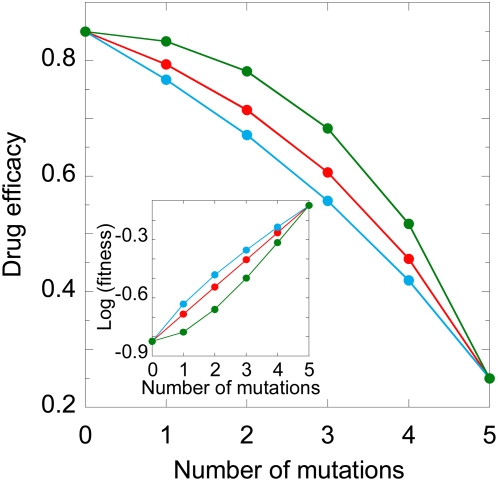
Efficacy, ε*_m_*, of a genome carrying *m* (0≤*m*≤*n*) resistance mutations, when the genetic barrier *n* = 5, ε_0_ = 0.85, ε*_n_* = 0.25, and the epistasis, *E* = 0.005 (green), 0 (red), and −0.005 (blue). The inset shows the corresponding fitness ( = 1−ε*_m_*) profiles.

**Figure 4:**
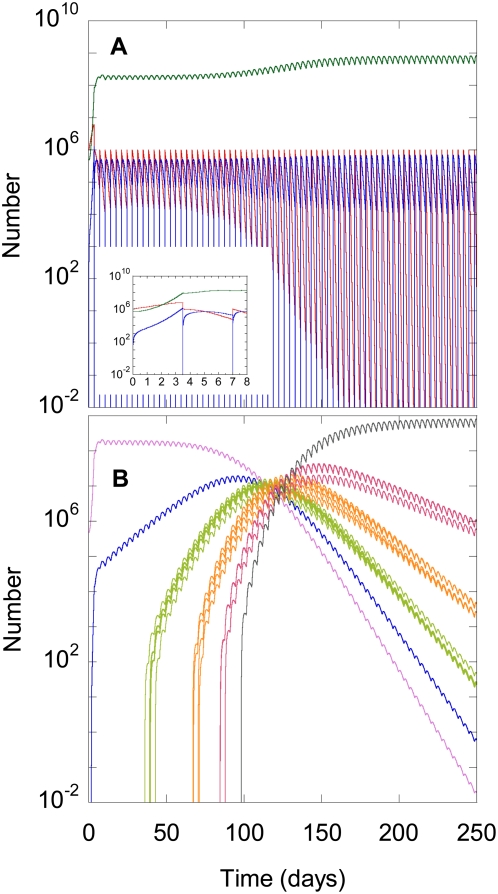
Model predictions of cell and viral dynamics. The time evolution of (A) the number of uninfected cells (red), infected cells (blue), and infectious virions (green) and (B) homozygous virions carrying wild-type genomes (pink) and single (blue), double (green), triple (orange), quadruple (red), and quintuple (black) mutants, obtained by solving Eqs. (1)–(9) with the parameters *T*_0_ = 10^6^ cells, *V*_00_ = 5×10^5^ virions, *n* = 5, *l* = 100 nucleotides, and ε*_m_* from Figure 3 with *E* = 0. The remaining parameters are listed in Methods. The inset in (A) shows the evolution for the first two passages.

We perform calculations for a genetic barrier *n* = 5, representative of ritonavir-boosted PIs [38]. We let the separation between successive resistance mutations, *l* = 100 nucleotides, and choose the efficacy profile shown in Figure 3 with epistasis *E* = 0 (also see Methods). Here, the efficacy against the wild type, ε_0_, and against the strain with *n* mutations, ε*_n_*, correspond to 400 nM of tipranavir [10]. We assume that the efficacy against intermediate mutants, ε*_m_*, depends on the number of mutations, *m* (0≤*m*≤*n*), the genomes contain. In Figure 4A, we present the evolution of populations of uninfected cells, *T*, infected cells, 
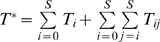
, and infectious virions, 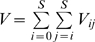, with time following the onset of the experiment. In the first passage, *T* rises due to the proliferation of uninfected cells (Figure 4A, inset). At the same time, T^*^ rises due to the infection of *T*, and *V* rises sharply due to viral production from T^*^. In the second passage, the higher *V* enhances the infection of *T*. Here, the loss of *T* due to infection dominates cell proliferation and *T* declines. Consequently, following an initial rise of T^*^ due to infection of *T*, target cell limitation lowers the formation of new infected cells and causes T^*^ to decline. The resulting lower viral production causes *V* to decline as well. This two phase behavior within a passage–an initial rise and the subsequent fall of T^*^–is observed in experiments [7] and is explained by models [37],[39]. The same two phase behavior repeats in ensuing passages and an oscillatory pseudo steady state is attained. Gradually, *V* rises marking the emergence of drug resistant genomes.

#### Emergence of resistant genomes

In Figure 4B, we present the time-evolution of populations of infectious homozygous virions containing genomes with different numbers of resistance mutations. Initially, the viral population contains the wild type genomes alone. Following the onset of infection, as *V* rises, the rate of formation of single mutants increases. Single mutants emerge here in the first passage. Because drug efficacy is lower against single mutants than against wild type genomes (ε_1_<ε_0_; Figure 3), single mutants begin to grow at the expense of the wild type. (We note that unlike the scenario *in vivo*, passage experiments are designed to allow the growth of even wild-type genomes in the initial passages.) As infections by single mutants become significant, the rate of formation of double mutants rises. Double mutants emerge in ∼40 days. With *n* = 5, 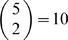 different double mutants are possible. They emerge at slightly different times because of the differential influence of recombination: A double mutant that contains the two mutations on adjacent resistance loci is less likely to be formed by recombination than a double mutant with mutations on well separated loci; the number of crossovers increases with the separation [40]. Again, because ε_2_<ε_1_<ε_0_ (Figure 3), double mutants begin to outgrow single mutants and the wild type. This process continues with the sequential emergence of higher mutants until by *W*∼100 days quintuple mutants emerge, which possess high level resistance to the drug. *W*∼100 days is thus the waiting time for the emergence of the genome that overcomes the genetic barrier of the drug. From this point on, quintuple mutants dominate the viral population.

Several characteristics of drugs, viz., the genetic barrier, *n*, epistasis, *E*, and the separation between adjacent resistance loci, *l*, influence *W*, which we examine next.

#### Effect of the genetic barrier

**Figure 5:**
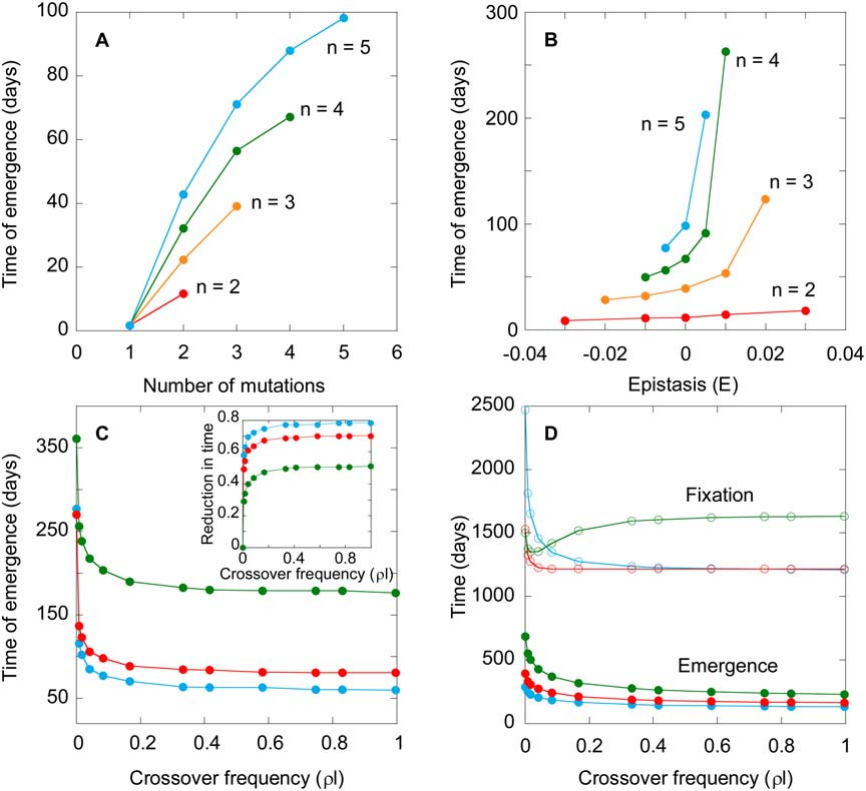
Model predictions of emergence and fixation times. The expected waiting time for the emergence of (A) genomes with different numbers of resistance mutations for different *n* when *E* = 0, (B) the corresponding *n* ^th^ mutants as a function of *E*, (C) quintuple mutants when *n* = 5 as a function of the crossover frequency (*ρl*), for *E* = 0.005 (green), 0 (red), −0.005 (blue). The inset in (C) shows the corresponding reduction in the time of emergence, 1−*W*(*ρl*)/*W*(*ρl* = 0). (D) Model predictions of emergence (filled symbols) and fixation (open symbols) times of double mutants when *n* = 2 and *E* = 0.05 (green), 0 (red), −0.05 (blue). In (A) to (C), we let ε_0_ = 0.85 and ε*_n_* = 0.25, whereas in (D) ε_0_ = 0.1 and ε*_n_* = 0. All the other parameters are identical to those in Figure 4.

To examine the influence of the genetic barrier, we vary *n* for fixed values of ε_0_, ε*_n_*, and *E*, and predict *W*. We find that *W* increases dramatically with *n*. For instance, *W* increases from ∼12 days when *n* = 2 to ∼100 days when *n* = 5 (Figure 5A), underscoring the advantage of a drug with a large *n*. As *n* increases, the number of mutations necessary for resistance increases. The number of replication cycles required to accumulate the necessary mutations increases correspondingly and delays the emergence of resistant genomes. The development of resistance is inhibited further by the delayed emergence of intermediate mutants. For the same ε_0_ and ε*_n_*, the incremental fitness advantage with each mutation decreases as *n* increases. The smaller this advantage, the longer it takes for the resolution of the competition between different mutants. Thus, following their emergence, double mutants take longer to outgrow single mutants when *n* = 4 than when *n* = 3. When the influence of recombination is weak, triple mutants emerge predominantly by mutation of double mutants. Consequently, the waiting time for the emergence of triple mutants is larger when *n* = 4 than when *n* = 3. Indeed, triple mutants emerge in ∼40 days when *n* = 3 and ∼55 days when *n* = 4 (Figure 5A). Thus, the increasing number of replication cycles required and the slower emergence of intermediate mutants together result in the dramatic increase of *W* with *n*.

#### Effect of epistasis

The increase of *W* with *n* is amplified when *E*>0. Whereas *W* increases from ∼12 to ∼100 days when *E* = 0, *W* increases from ∼12 to ∼205 days when *E* = 0.005 as *n* increases from 2 to 5 (Figure 5B). As *E* increases, the fitness of intermediate mutants decreases (Figure 3). Consequently, intermediate mutants emerge slower, increasing *W* (Figures S1 and 5B). In contrast, the fitness of intermediate mutants is higher (Figure 3) and hence the increase of *W* with *n* is suppressed when *E*<0 (Figures S1 and 5B).

#### Effect of recombination

Interestingly, recombination decreases *W* regardless of *E* (Figure 5C). Recombination accelerates the accumulation of mutations and expedites the emergence of resistant strains. Thus, upon increasing the recombination rate, which we accomplish by increasing *l*, the separation between resistance loci, *W* drops by ∼50% when *E* = 0.005 and by ∼80% when *E* = −0.005 for *n* = 5 from that in the absence of recombination (Figure 5C, inset). The greater drop in *W* when *E*<0 is because of the increased fitness (Figure 3) and hence greater prevalence of intermediate mutants, which in turn enhances the likelihood of the formation of heterozygous virions and facilitates the accumulation of mutations by recombination. This influence of recombination on *W* is robust to changes in *n* (Figure S2).

That recombination invariably lowers *W* is intriguing given that several studies argue that recombination may inhibit the fixation of resistance when *E*>0 (e.g., see [27],[33]). We therefore compute the fixation time, *F*, defined as the time when 90% of the genomes in the viral population are *n* ^th^ mutants. We find interestingly that recombination increases *F* when *E*>0, consistent with current expectations (Figure 5D) [27],[33]. When *n* = 2, *W* marks the time when the first double mutant emerges in the viral population. For times smaller than *W*, the wild-type and the single mutants alone exist in the viral population. Recombination then brings the mutations on the two single mutants together and accelerates the emergence of the double mutant regardless of *E*. After the double mutant emerges, recombination influences the competitive dynamics of the different viral strains and alters *F*. In particular, not only do single mutants recombine to yield the double mutant, but the double mutant could also be lost by recombination with the wild type. When *E*>0, recombination tends to lower the prevalence of the double mutant [27], resulting in the observed increase in *F*. Thus, recombination may lower *W* and yet increase *F*. We note that the distinction between emergence and fixation has been recognized earlier [41]. When *E*<0, recombination enhances the prevalence of the double mutant and decreases *F*. We recognize here that the influence of recombination on *F* is determined not only by *E* but also by *n*, ε_0_, ε*_n_*, and the population size of HIV and its variation, examining all of which is beyond the scope of the present study. Our aim here is to predict *W*, which marks the emergence of drug resistant genomes.

### Comparison with Experiments

**Figure 6:**
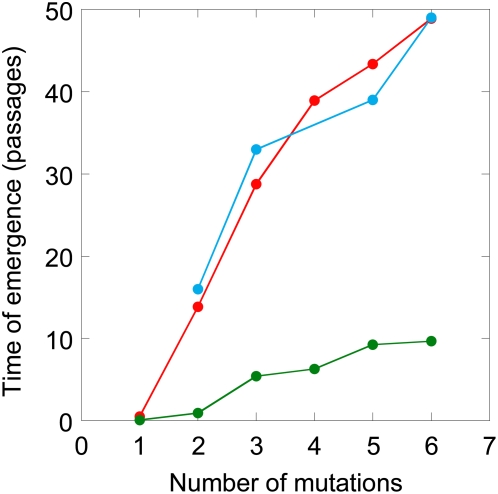
Comparison of model predictions (red) and the experimentally observed [10] (blue) times of emergence of different mutants resistant to tipranavir. The different mutants and the corresponding *IC*_50_ values are listed in Table S1. Also shown are the times when the numbers of the different mutant proviruses first reach 1 (green) predicted by our model when, following current models [34],[35], we assume that the waiting times *w_i_* = 0.

We apply our model to describe the development of resistance to tipranavir in *in vitro* serial passage experiments [10]. We let *n* = 6 because a genome with 6 resistance mutations exhibited >10 fold resistance to tipranavir in these experiments. We choose *IC*_50_ values for different intermediate mutants from the ranges determined experimentally (Table S1). Further, we employ actual distances between resistance sites to calculate the recombination probabilities and also assign fitness advantages to genomes containing specific combinations of mutations (Table S1). (In contrast, in our calculations above, the number of mutations and not their specific combinations was assumed to determine the fitness advantage.) We also vary the concentration of tipranavir as in the experiments (Table S2). Further, following the experimental protocol, we employ 90% of the viral population at the end of any passage to initiate infection in the succeeding passage when the drug concentration is maintained constant across the passages and 50% of the viral population when the drug concentration is increased in the succeeding passage. Genomes carrying 2, 3, 5 and 6 resistance mutations were first observed in the experiment in passages 16, 33, 39 and 49, respectively [10]. In close agreement, our model predicts the emergence of these genomes in passages 14, 29, 44 and 49, respectively (Figure 6). (Ignoring the concept of the waiting time, i.e., letting *w_i_* = 0 in our model, severely underpredicts the times of emergence of drug resistant genomes (Figure 6). The agreement between model predictions and experiments indicates that our model captures the underlying dynamics of the development of resistance to antiretroviral drugs accurately.

## Discussion

Current models of HIV dynamics successfully predict short-term changes in the plasma viral load in patients undergoing therapy but fail to provide a quantitative description of the emergence of drug resistance [34]–[36]. A key limitation of current models is the underlying assumption that the emergence of resistant genomes is governed by deterministic effects. Deterministic effects predominate when the population of cells in an infected individual is large. In a finite cell population, because the probability of the formation of a resistant genome with many mutations can be small, resistant genomes emerge stochastically. The waiting time for the emergence of resistant genomes can therefore be substantial. In contrast, by assuming that deterministic effects predominate, current models predict that resistant genomes emerge, albeit in very small numbers, immediately upon the onset of therapy. Once resistant genomes emerge, their numbers grow due to viral production from the cells they infect leading to the rapid fixation of resistance. Current models thus underestimate the time for the development of drug resistance (Figure 6).

Simulations of viral evolution, based on models of population genetics, consider finite populations and present descriptions of the stochastic emergence of drug resistant genomes [28],[29],[32]. Importantly, the simulations also enable incorporation of recombination and fitness interactions between multiple loci, which are central to the development of drug resistance but are not easily incorporated in models of HIV dynamics. The simulations, however, make several simplifying assumptions, such as fixed population sizes and discrete generations, which approximate the dynamics of the development of drug resistance and introduce uncertainties in the influence of underlying processes, such as recombination [30],[33]. Besides, simulations are difficult to incorporate in mathematical formalisms for therapy optimization.

Here, we develop a model that employs the deterministic framework of models of HIV dynamics and at the same time captures the influence of stochastic effects associated with the emergence of drug resistant genomes. To accomplish this, we invoke the concept of the expected waiting time. We develop a detailed description of mutation and recombination between multiple loci, which enables calculation of the probability of the formation of resistant genomes in one replication event. Given the viral and cell populations and the efficacy of the drug, the frequency of replication events and hence the rate of formation of resistant genomes is determined. From the rate of formation, we estimate the expected waiting time for the first resistant genome to emerge. Different mutant genomes are assumed to appear first in the viral population at their respective expected waiting times. The limitation of current models of HIV dynamics, which predict the emergence of resistant genomes immediately upon the start of therapy, is thus overcome. Yet, by calculating the “expected” waiting time, our model captures the influence of stochastic effects associated with the emergence of resistant genomes in an averaged sense and retains the dynamical framework of current models. The limitations of population genetics based simulations are also thus overcome.

The waiting time for the emergence of a genome carrying a certain number of mutations depends on the times of emergence and the growth of subpopulations of genomes with fewer mutations. Our model assumes that the latter genomes emerge at their expected waiting times. Consequently, the variation in the waiting times for the emergence of higher mutants due to the variation in the times of emergence of lower mutants is suppressed in our model. Further, following emergence, particularly when the population size is small, the chance that stochastic forces cause the extinction of genomes may be significant. We assume, however, that following emergence, the growth of genomes is deterministic. The extent of the uncertainties introduced in our model predictions by these simplifying assumptions remains to be estimated. Semi-stochastic simulations, where the times of emergence of mutant genomes alone are determined stochastically, and fully stochastic simulations (see, e.g., [42]) of the emergence of mutant genomes would serve as tests of our model. Performing the simulations, however, is beyond the scope of the present study. Here, we compare model predictions with experiments and find that our predictions are in close agreement with experimental observations [10] of the times of emergence of various genomes possessing different degrees of resistance to tipranavir, suggesting that our model captures the underlying dynamics of the development of drug resistance by HIV.

Model predictions indicate that the waiting time, *W*, for the emergence of the strain that overcomes the genetic barrier of a drug depends on several factors that may be tuned during drug design. A large genetic barrier significantly enhances *W*. This enhancement of *W* with the genetic barrier is amplified when fitness interactions between resistance loci exhibit positive epistasis. Recombination, in contrast, lowers *W* regardless of epistasis or the genetic barrier. If the separation between resistance loci is small, however, the role of recombination is suppressed. Thus, for delaying the emergence of resistant genomes, drugs that offer large genetic barriers with resistance sites localized tightly on the viral genome and exhibiting positive epistatic interactions are desirable. These observations may serve as guidelines for structure-based drug design [43]. The fixation of resistant genomes following their emergence may depend differently on drug characteristics and remains to be fully elucidated.

When distinctions between different viral genomes are ignored, the expected waiting time vanishes and our model reduces to the basic model of HIV dynamics, which successfully captures viral load changes in patients undergoing therapy [3],[34]. Our model may thus be applied to predict drug failure *in vivo*. Several advances of our model are essential, however, to describe the *in vivo* scenario accurately. First, the higher frequency of multiple infections [44], possible cell-cell transmission of infection [45],[46], and the existence of resistance mutations prior to the onset of therapy [1] *in vivo* must be incorporated into our model. Second, during potent drug therapy, viral replication may be suppressed significantly, resulting in a small effective population size of HIV. The variation of the waiting time about the mean may then become large. Consequently, the assumption that mutant genomes emerge at their expected waiting times becomes less accurate. Our model must therefore be advanced to account for the variation of the emergence times of genomes *in vivo*. Third, our model must be extended to drugs from other drug classes to mimic current combination therapies. With these advances, our model would enable timing the emergence of resistance to drugs *in vivo* and facilitate the identification of treatment protocols that maximally impede the failure of current therapies.

## Methods

### Dynamical Equations

We present equations below that describe the *in vitro* dynamics of various cell and viral populations.

#### Uninfected cells



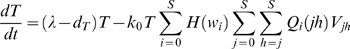
(1)Uninfected cells, *T*, proliferate at rate *λ* and die at rate *d_T_*. The rate of formation of infected cells *T_i_* containing genome *i* is 
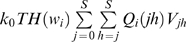
 (see below). Summation over *i* from 0 to *S* = 2*^n^*−1 yields the total rate of loss of *T* due to infection by free virions. At the beginning of each passage, *T* is set to *T*_0_ = 10^6^.

#### Singly infected cells



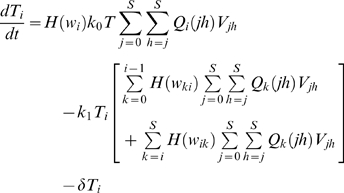
(2)where *i* ∈ {0, 1, 2…*S*}. Here, *k*_0_ *TV_jh_* is the rate at which virions *V_jh_* infect *T*. Following infection, the genomes *j* and *h* undergo reverse transcription to produce provirus *i* with the probability *Q_i_*(*jh*) (see below). Thus, *k*_0_ *TQ_i_*(*jh*)*V_jh_* is the rate at which uninfected cells acquire genome *i* following infection by *V_jh_*. Summation over *j* and *h* yields the total rate, 
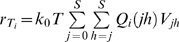
, of the production of cells *T_i_*.

The rate 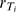 can be small, especially if genome *i* contains many mutations. Consequently, when the cell population is finite, the formation of the first cell *T_i_* is stochastic. We define *t_i_* as the waiting time for the formation of the first cell *T_i_*. *t_i_* may assume any value between 0 and ∞ with a probability density dependent on 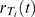 (see below). Here, we assume as a simplification that the first cell *T_i_* emerges at the expected waiting time 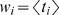. In addition, we assume that following emergence, the growth of *T_i_* is deterministic. We therefore multiply the rate 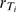 in Eq. (2) with the Heaviside function,
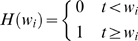
(3)so that *T_i_* = 0 when *t*<*w_i_*, and cells *T_i_* are produced at the rate 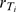 when *t*≥*w_i_*. By allowing *T_i_* to grow at the rate 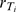 from time *t* = 0, extant models of HIV dynamics underestimate the time of emergence of drug resistant genomes. We derive estimates of *w_i_* below. We solve Eq. (2) with the initial condition, *T_i_*(*w_i_*) = 1, and reset *T_i_* to zero at the start of every passage. The other two terms in Eq. (2) represent the loss of cells *T_i_* due to death at rate *δ* and due to further infections, which convert *T_i_* to doubly infected cells.

#### Doubly infected cells



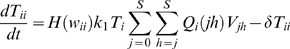
(4a)Here 
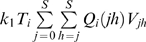
 is the rate at which cells *T_i_* acquire a second provirus *i*. *w_ii_* is the expected waiting time for the emergence of the first cell *T_ii_*. After the first infection of a cell, down-modulation of cell surface CD4 receptors reduces the susceptibility of the cell to new infections [47],[48]. Here, we let *k*_1_(<*k*_0_) be the mean rate constant for the infection of singly infected cells [37].

For cells infected with two different kinds of proviruses, we write
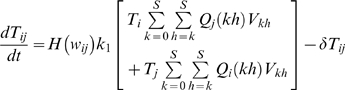
(4b)where *i*<*j* and *i*, *j* ∈ {0, 1, 2…*S*}. Here, the two terms in the brackets correspond to the two ways of forming a doubly infected cell *T_ij_*: a cell *T_i_* can be infected by provirus *j* or a cell *T_j_* by provirus *i*. Following earlier studies, we ignore more than two infections of cells [37]. We solve the above equations with the initial conditions *T_ii_*(*w_ii_*) = 1 and *T_ij_*(*w_ij_*) = 1, respectively, and reset *T_ii_* and *T_ij_* to zero at the start of every passage.

*Waiting time*: At any time *t*, the rate of formation of cells *T_i_*,
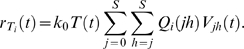
(5)Because individual infection events occur independently, the formation of *T_i_* may be described as a Poisson process with the instantaneous rate 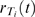. The probability that the waiting time, *t_i_*, for the first formation of a cell *T_i_* is smaller than *s* is then 
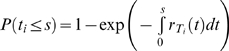
, 0≤*s*<∞ [49]. It follows that the expected waiting time, 
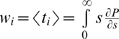
, or
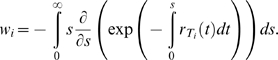
(6)Similarly,
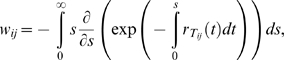
(7)where 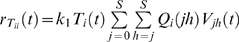 and when *i*<*j*, 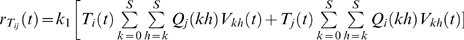.

We recognize that the evaluation of the waiting times requires knowledge of the rates, e.g., 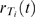, at all times *t*. We therefore devise a numerical approximation to estimate *w_i_* based on the values of 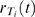 until a given time *t*, which allows explicit integration of the dynamical equations (Text S1).

#### Reverse transcription

To evaluate the probability *Q_i_*(*jh*), we decouple mutation and recombination [29],[32],[50]. We let genomes *j* and *h* recombine to produce genome *k* with probability *R_k_*(*jh*) and let genome *k* mutate to genome *i* with probability *P_ik_* [50]. The number of different recombinants *k* that can be produced is 2*^d^*, where *d*≤*n* is the number of sites at which *j* and *h* differ. Summing over all the recombinants *k* gives the total probability of producing genome *i* by reverse transcription of genomes *j* and *h*,
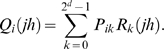
(8)


#### Recombination

To determine *R_k_*(*jh*), we compare genomes *j* and *h* at each of the *n* drug resistance sites and identify the distances *l*_1_, *l*_2_, etc., between the *d* successive sites at which the genomes differ (Figure S3). We then compare genome *k* with the genomes *j* and *h* to determine on which genome, *j* or *h*, the enzyme reverse transcriptase (RT) must be at each of the *d* distinguishing sites in order to yield the genome *k*. Figure S3 illustrates the desired path of RT for given *j*, *h*, and *k*. If *P_des_*(*m*) is the probability that RT is on the desired genome at the *m* ^th^ distinctive site, then 
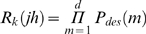
, which is readily evaluated based on the probabilities that RT undergoes odd and even crossovers in any length *l* (Figure S3).

#### Mutation

To calculate the probability of mutation, *P_ik_*, we compare the two genomes *i* and *k* at the *n* drug resistance sites and identify the *u* sites where the two genomes differ. The probability that genome *k* mutates at these *u* sites alone is 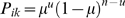, where 0≤*u*≤*n* and *μ* is the mutation rate.

#### Virions



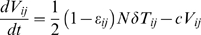
(9a)

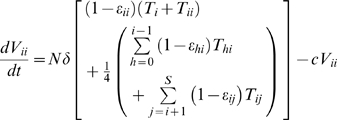
(9b)

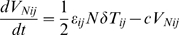
(9c)

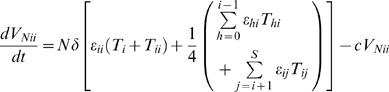
(9d)Here *V_ii_* and *V_ij_* denote infectious and 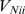 and 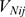 non-infectious virions. In the absence of the drug, cells *T_i_* and *T_ii_* produce homozygous virions *V_ii_* and cells *T_ij_* produce both homozygous virions *V_ii_* and *V_jj_* and heterozygous virions *V_ij_* in the proportion ¼, ¼, and ½, respectively [37]. All infected cells with at least one provirus of type *i* thus contribute to the production of *V_ii_*. 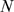 is the viral burst size, *δ* is the death rate of infected cells and *c* is the clearance rate of free virions. The above equations are solved with the initial condition that the wild type virions *V*_00_ alone exist at the start of the first passage. For every subsequent passage, the free virions at the end of the previous passage are employed to initiate infection.

#### Drug efficacy

The efficacy of a PI is the fraction of progeny virions that it renders non-infectious. We assume that the drug efficacy ε*_ii_* against the protease produced by genome *i* depends on the number of resistance mutations, *m*(*i*), the genome contains, i.e., ε*_ii_* = ε*_m_* [23]. We fix the efficacy against the wild type, ε_0_, and that against the strain with *n* mutations, ε*_n_*, and determine the efficacies against intermediate strains from the epistasis, *E*, which is assumed constant for every pair of loci differing by two resistance mutations [27]. If *E* = 0, then the mutations do not interact with each other and each additional mutation increases the log fitness, which we define as log(1−ε*_m_*), by the same amount (Figure 3). When *E*<0 (>0), the mutations interact antagonistically (synergistically) in increasing log fitness. For cells *T_i_* and *T_ii_*, the fraction of progeny virions rendered noninfectious is ε*_ii_*. For cells *T_ij_* containing distinct proviruses, phenotypic mixing [27] implies that proteases *i* and *j* are equally likely to be included in a budding virion so that, on average, the fraction of virions rendered noninfectious is ε*_ij_* = (ε*_ii_*+ε*_jj_*)/2.

When the efficacy is determined as a function of the drug concentration, *C*, as in our calculations in Figure 6, we write ε*_ii_* = *C*/(*IC*_50_(*i*)+*C*), where *IC*_50_(*i*) is the value of *C* at which ε*_ii_* is 50% [51].

Equations (1) to (9) represent a model of HIV dynamics that describes the development of resistance to a PI with a genetic barrier *n*. We solve the equations using a computer program written in C.

### Model Parameters

We employ the following parameter values based on earlier studies [4],[37],[39],[52]: the birth and death rate of uninfected T cells, *λ* = 0.624 day^−1^ and *d_T_* = 0.018 day^−1^; the death rate of infected cells, *δ* = 1.44 day^−1^; the viral burst size, 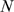 = 10^3^; the viral clearance rate, *c* = 0.35 day^−1^; the second order rate constants of the infection of uninfected and singly infected cells, *k*_0_ = 10^−8^ day^−1^ and *k*_1_ = 0.7*k*_0_; the mutation and recombination rates, *μ* = 3×10^−5^ per site per replication, and *ρ* = 8.3×10^−4^ crossovers per site per replication.

## Supporting Information

Figure S1 Model predictions of the times of emergence of various mutants as the genetic barrier varies from *n* = 2 to 4 and the epistasis *E* = −0.01 (A) and 0.01 (B). All the other parameters are identical to those in Figure 4.
(0.06 MB DOC)

Figure S2 Model predictions of the time of emergence of nth mutants for different epistatic interactions, *E*, and genetic barriers *n* = 3 (A) and *n* = 4 (B). All the other parameters are identical to those in Figure 4.
(0.06 MB DOC)

Figure S3 Schematic representation of the production of genome *k* by recombination of genomes *j* and *h*. Stars indicate mutations. The arrow marks the desired path of the enzyme reverse transcriptase (RT) and allows determination of the probability, *R_k_(jh)*, that genome *k* is formed. At the first site where *j* and *h* differ, the probability that RT is on the desired genome, *P_des_(1)*, is 1/2, because reverse transcription can commence on either of the two genomes with equal likelihood. At the second site, if the desired genome is the same as that of the first site, then RT will be on the desired genome if it undergoes an even number of crossovers in the intervening distance *l*_1_, the probability of which we write as *P_des_(2) = P_even_(l_1_)*. If the desired genome is different from that at the first site, then the probability that RT will be on the desired genome is *P_des_(2) = P_odd_(l_1_)*. It follows that *R_k_(jh)* = Π*P_des_(m)*, where *m* ranges from 1 to *d* and the probabilities that even and odd crossovers occur in length l are [37] *P_even_(l) = exp(−ρl)cosh(ρl)* and *P_odd_(l) = exp(−ρl)sinh(ρl)*, respectively, with ρ the per site recombination rate of HIV.
(0.03 MB DOC)

Table S1 Sequences resistant to tipranavir and their *IC*_50_ values. Sequences with different combinations of resistance mutations observed experimentally, corresponding binary sequences illustrating the specific locations of mutations, marked as 1, when *n* = 6, and the respective *IC*_50_ values employed in our model are listed. The experimental *IC*_50_ values [10] are in brackets. In our simulations (Figure 6), we assign *IC*_50_ values to genomes as follows. To each genome *i*, we assign an *IC*_50_ value equal to the *IC*_50_ of the genome below that has the maximum number of mutations in common with the genome *i* but has no mutations in addition to those contained in *i*. For instance, the genome 101001 is assigned an *IC*_50_ of 101 nM, whereas the genome 000110 is assigned an *IC*_50_ of 60 nM, equal to the wild-type.
(0.04 MB DOC)

Table S2 Drug concentrations employed in the experiments [10] and in our calculations of Figure 6.
(0.03 MB DOC)

Text S1 Estimates of waiting times
(0.06 MB DOC)
