# Supplementary material for: Timing the Emergence of Resistance to Anti-HIV Drugs with Large Genetic Barriers
Source: PLoS Comput Biol. 2009 Mar 13;5(3):e1000305. doi: 10.1371/journal.pcbi.1000305 (PMC2643484; doi:10.1371/journal.pcbi.1000305)
Supplement: Figure S3 — Schematic representation of the production of genome k by recombination of genomes j and h. Stars indicate mutations. The arrow marks the desired path of the enzyme reverse transcriptase (RT) and allows determination of the probability, Rk(jh), that genome k is formed. At the first site where j and h differ, the probability that RT is on the desired genome, Pdes(1), is 1/2, because reverse transcription can commence on either of the two genomes with equal likelihood. At the second site, if the desired genome is the same as that of the first site, then RT will be on the desired genome if it undergoes an even number of crossovers in the intervening distance l 1, the probability of which we write as Pdes(2) = Peven(l1). If the desired genome is different from that at the first site, then the probability that RT will be on the desired genome is Pdes(2) = Podd(l1). It follows that Rk(jh) = ΠPdes(m), where m ranges from 1 to d and the probabilities that even and odd crossovers occur in length l are [37] Peven(l) = exp(−ρl)cosh(ρl) and Podd(l) = exp(−ρl)sinh(ρl), respectively, with ρ the per site recombination rate of HIV. (0.03 MB DOC) [file pcbi.1000305.s003.doc]

**§**

*l*_1_

*l*_2_

*l*_3_

*j*

*h*

*k*
