# Supplementary material for: Timing the Emergence of Resistance to Anti-HIV Drugs with Large Genetic Barriers
Source: PLoS Comput Biol. 2009 Mar 13;5(3):e1000305. doi: 10.1371/journal.pcbi.1000305 (PMC2643484; doi:10.1371/journal.pcbi.1000305)
Supplement: Table S1 — Sequences resistant to tipranavir and their IC 50 values. Sequences with different combinations of resistance mutations observed experimentally, corresponding binary sequences illustrating the specific locations of mutations, marked as 1, when n = 6, and the respective IC 50 values employed in our model are listed. The experimental IC 50 values [10] are in brackets. In our simulations (Figure 6), we assign IC 50 values to genomes as follows. To each genome i, we assign an IC 50 value equal to the IC 50 of the genome below that has the maximum number of mutations in common with the genome i but has no mutations in addition to those contained in i. For instance, the genome 101001 is assigned an IC 50 of 101 nM, whereas the genome 000110 is assigned an IC 50 of 60 nM, equal to the wild-type. (0.04 MB DOC) [file pcbi.1000305.s004.doc]

| Number of mutations | Mutations in sequences observed experimentally [10] | Corresponding binary sequences | IC_50_(nM) |
| --- | --- | --- | --- |
| 0 | - | 000000 | 60 (60±13) |
| 1* | L33F  I84V | 001000  000001 | 78 |
| 2 | L33F, I84V | 001001 | 101 (101±43) |
| 3 | L33F, K45I, I84V | 001101 | 164 (124±43) |
| 4* | I13V, L33F, K45I, I84V  V32I, L33F, K45I, I84V | 101101  011101 | 400 |
| 5 | I13V, V32I, L33F, K45I, I84V | 111101 | 450 (407±206) |
| 6 | I13V, V32I, L33F, K45I, V82L, I84V | 111111 | 967 (967±434) |

*These mutants were not observed in the experiments [10].
