# Supplementary material for: Timing the Emergence of Resistance to Anti-HIV Drugs with Large Genetic Barriers
Source: PLoS Comput Biol. 2009 Mar 13;5(3):e1000305. doi: 10.1371/journal.pcbi.1000305 (PMC2643484; doi:10.1371/journal.pcbi.1000305)
Supplement: Table S2 — Drug concentrations employed in the experiments [10] and in our calculations of Figure 6. (0.03 MB DOC) [file pcbi.1000305.s005.doc]

| Passage number | Tipranavir concentration (nM) |
| --- | --- |
| 1-12 | 400 |
| 13-18 | 800 |
| 19-38 | 1000 |
| 39-48 | 2000 |
| 49-52 | 5000 |
