# Supplementary material for: Timing the Emergence of Resistance to Anti-HIV Drugs with Large Genetic Barriers
Source: PLoS Comput Biol. 2009 Mar 13;5(3):e1000305. doi: 10.1371/journal.pcbi.1000305 (PMC2643484; doi:10.1371/journal.pcbi.1000305)
Supplement: Text S1 — Estimates of waiting times (0.06 MB DOC) [file pcbi.1000305.s006.doc]

**Estimates of waiting times**

The expected waiting time, *w_i_*, for the emergence of cells *T_i_*, can be determined using Eq. (6) if $r_{T_{i}}\left(t\right)$ is known for all *t*. $r_{T_{i}}\left(t\right)$, however, depends on *w_i_*,: $r_{T_{i}}\left(t\right)$ is small for *t* < *w_i_* because *T_i_*(*t* < *w_i_*) = 0 and cells *T_i_* must be produced by mutation and/or recombination of other genomes, whereas $r_{T_{i}}\left(t\right)$ increases drastically for *t* > *w_i_* because of viral production from cells *T_i_* and subsequent infections. Model equations are thus strongly coupled and difficult to solve. To overcome this limitation, we adopt the following numerical approximation.

We recognize that during the numerical integration of the dynamical equations, $r_{T_{i}}\left(t\right)$ is known until the present integration step *q*, or time *t* = *q*Δ*t*, where Δ*t* is the time step of integration. As an approximation, we assume that $r_{T_{i}}\left(t>q\Delta t\right)=r_{T_{i}}\left(q\Delta t\right)$. We can then evaluate the integral in Eq. (6) and obtain an estimate of *w_i_*, which we denote ξ*_i_*(*q*Δ*t*).

At the first time step, we write

$r_{T_{i}}\left(t\right)=r_{T_{i}}\left(0\right)$, (S1)

so that

$\xi_{i}\left(0\right)=\int_{s=0}^{\infty} s\frac{\partial}{\partial s}\left(-\exp\left(-r_{T_{i}}\left(0\right)s\right)\right)ds=\int_{s=0}^{\infty} r_{T_{i}}\left(0\right)s\exp\left(-r_{T_{i}}\left(0\right)s\right)ds=\frac{1}{r_{T_{i}}\left(0\right)}$ (S2)

where $r_{T_{i}}\left(0\right)=k_{0}T\left(0\right)\sum_{j=0}^{S} \sum_{h=j}^{S} Q_{i}\left(jh\right)V_{jh}\left(0\right)$ is the initial rate of formation of cells *T_i_* (Eq. 5)
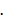
 At the second time step, we write,

$r_{T_{i}}\left(t\right)=\left\{\begin{matrix} r_{T_{i}}\left(0\right) & t<\Delta t \\ r_{T_{i}}\left(\Delta t\right) & t\geq\Delta t. \end{matrix}\right.$ (S3)

The expected waiting time is then estimated to be

$\begin{matrix} \xi_{i}\left(\Delta t\right)=\int_{s=0}^{\Delta t} s\frac{\partial}{\partial s}\left(-\exp\left(-r_{T_{i}}\left(0\right)s\right)\right)ds+\int_{\Delta t}^{\infty} s\frac{\partial}{\partial s}\left(-\exp\left(-r_{T_{i}}\left(0\right)\Delta t\right)\exp\left(-r_{T_{i}}\left(\Delta t\right)\left(s-\Delta t\right)\right)\right)ds \\ =\frac{1}{r_{T_{i}}\left(0\right)}+\exp\left(-r_{T_{i}}\left(0\right)\Delta t\right)\left(\frac{1}{r_{T_{i}}\left(\Delta t\right)}-\frac{1}{r_{T_{i}}\left(0\right)}\right) \end{matrix}$ (S4)

Proceeding similarly, we obtain

$\begin{matrix} \xi_{i}\left(q\Delta t\right)=\int_{s=0}^{\Delta t} s\frac{\partial}{\partial s}\left(-\exp\left(-r_{T_{i}}\left(0\right)s\right)\right)ds+\int_{\Delta t}^{2\Delta t} s\frac{\partial}{\partial s}\left(-\exp\left(-r_{T_{i}}\left(0\right)\Delta t\right)\exp\left(-r_{T_{i}}\left(\Delta t\right)\left(s-\Delta t\right)\right)\right)ds+... \\ +\int_{q\Delta t}^{\infty} s\frac{\partial}{\partial s}\left(-\exp\left(-r_{T_{i}}\left(q\Delta t\right)\left(s-q\Delta t\right)\right)\prod_{j=0}^{q-1} \exp\left(-r_{T_{i}}\left(j\Delta t\right)\Delta t\right)\right)ds \\ =\frac{1}{r_{T_{i}}\left(0\right)}+\sum_{j=1}^{q} \left[\left(\frac{1}{r_{T_{i}}\left(j\Delta t\right)}-\frac{1}{r_{T_{i}}\left(\left(j-1\right)\Delta t\right)}\right)\exp\left(-\Delta t\sum_{k=0}^{j-1} r_{T_{i}}\left(k\Delta t\right)\right)\right] \end{matrix}$ (S5)

We compare this estimated waiting time, ξ*_i_*(*q*Δ*t*), with the time following the start of the ongoing passage, *q*Δ*t*. That value of *q* when ξ*_i_*(*q*Δ*t*) first becomes less than *q*Δ*t* is assumed to give the waiting time *w_i_* for the emergence of *T_i_*. In other words, if

$\xi_{i}\left(\left(q-1\right)\Delta t\right)>\left(q-1\right)\Delta t$ and $\xi_{i}\left(q\Delta t\right)\leq q\Delta t$ (S6)

then

$w_{i}=q\Delta t$. (S7)

The above description of *w_i_* applies when infection proceeds in a single (arbitrarily long) passage. With the start of every new passage, however, a fresh set of infection events begins. We therefore reset *q* to 0 at the start of every passage for those genomes *i* that have not emerged in the previous passages and recalculate ξ*_i_*(*q*Δ*t*), where *q* extends from 0 to *t_p_*/Δ*t* with *t_p_* the duration of a passage. Then, if the criterion (S6) is satisfied in passage *P*, we write *w_i_*=(*P*–1)*t_p_*+*q*Δ*t*. Waiting times for doubly infected cells are obtained in an analogous manner.
