# Supplementary material for: Timing the Emergence of Resistance to Anti-HIV Drugs with Large Genetic Barriers
Source: PLoS Comput Biol. 2009 Mar 13;5(3):e1000305. doi: 10.1371/journal.pcbi.1000305 (PMC2643484; doi:10.1371/journal.pcbi.1000305)
Supplement: Supplementary file 5 [file pcbi.1000305.s001.doc]

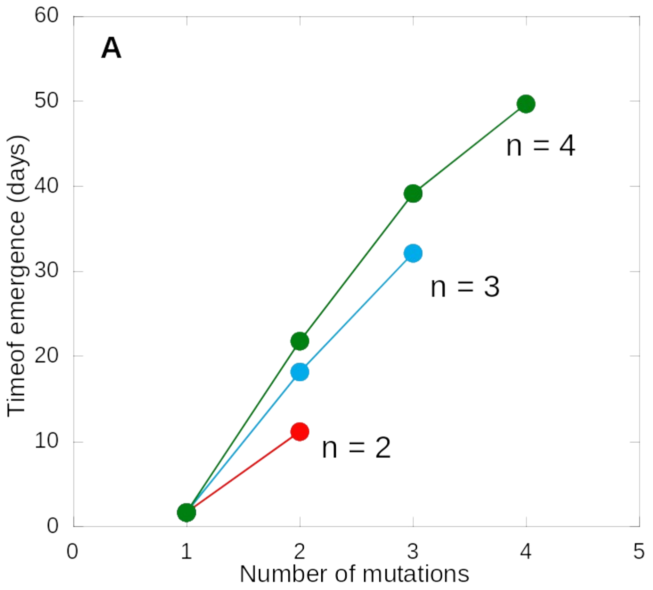
A
Timeof emergence (days)
Number of mutations
n = 4
n = 3
n = 2
60
50
40
30
20
10
0
0
1
2
3
4
5

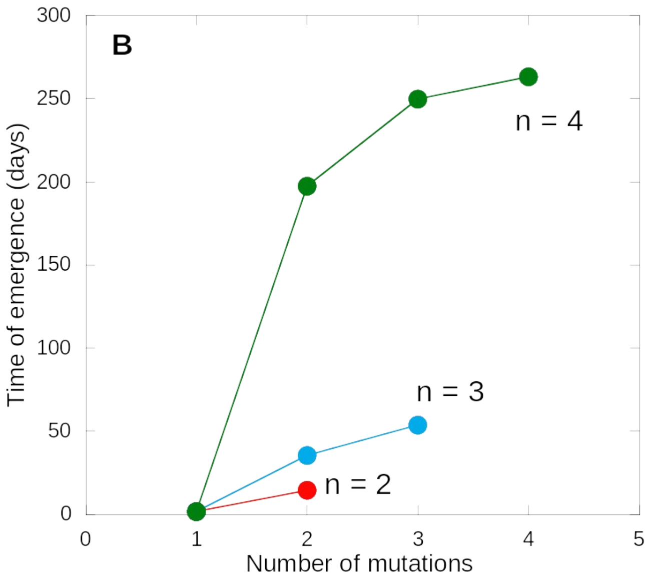
B
Time of emergence (days)
Number of mutations
n = 4
n = 3
n = 2
300
250
200
150
100
50
0
0
1
2
3
4
5
