# Supplementary material for: Timing the Emergence of Resistance to Anti-HIV Drugs with Large Genetic Barriers
Source: PLoS Comput Biol. 2009 Mar 13;5(3):e1000305. doi: 10.1371/journal.pcbi.1000305 (PMC2643484; doi:10.1371/journal.pcbi.1000305)
Supplement: Supplementary file 6 [file pcbi.1000305.s002.doc]

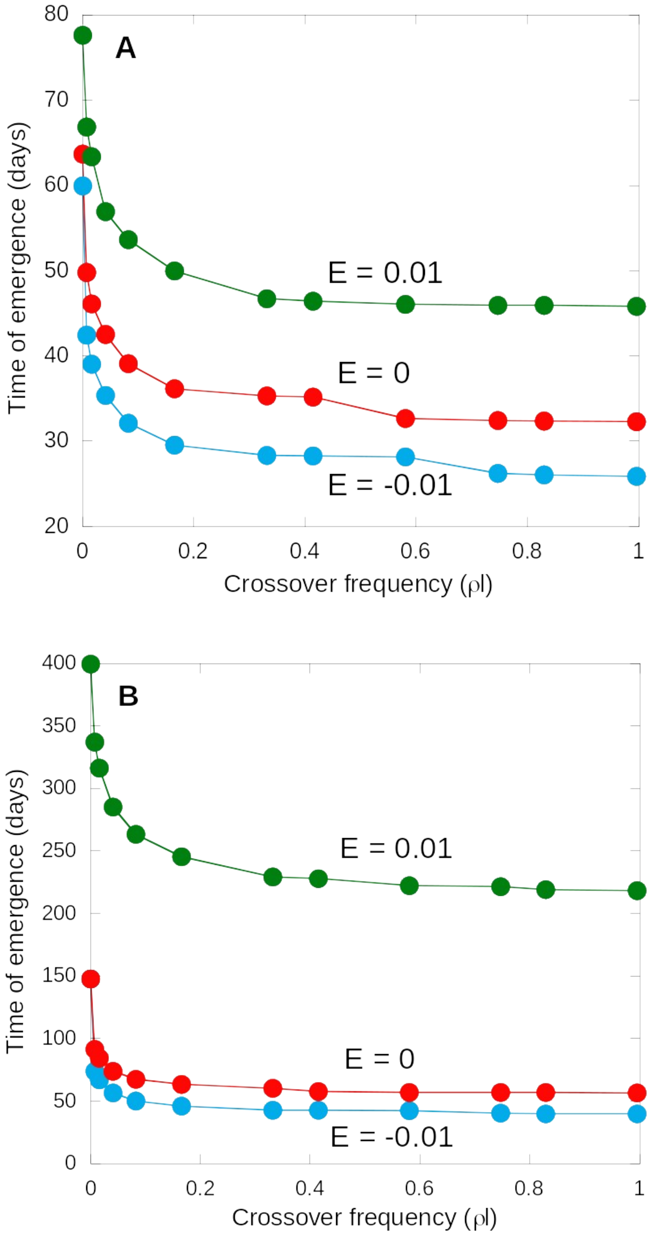

A
Time of emergence (days)
80
70
60
50
40
30
20
E = 0.01
E = 0
E = -0.01
0
0.2
0.4
0.6
0.8
1
Crossover frequency (ρl)
B
Time of emergence (days)
400
350
300
250
200
150
100
50
0
E = 0.01
E = 0
E = -0.01
0
0.2
0.4
0.6
0.8
1
Crossover frequency (ρl)
